# Micro-Crack Induced Buckypaper/PI Tape Hybrid Sensors with Enhanced and Tunable Piezo-Resistive Properties

**DOI:** 10.1038/s41598-019-53222-1

**Published:** 2019-11-15

**Authors:** Mustafa Danish, Sida Luo

**Affiliations:** Beihang University, School of Mechanical Engineering & Automation, Beijing, 100191 China

**Keywords:** Carbon nanotubes and fullerenes, Sensors and biosensors

## Abstract

Piezoresistive properties play a vital role in the development of sensor for structural health monitoring (SHM) applications. Novel stable crack initiation method (SCIM) is established to improve the gauge factor (GF) with maximum achievable working strain region for PI tape enabled buckypaper hybrid sensors. Cracks are generated by applying strain rate-controlled tension force using dynamic mechanical analyzer (DMA). The sensor has been cycled in tension to characterize GF with crack opening. It is determined experimentally that GF increases with increasing crack opening and crack becomes unstable when opening increases above 8 µm. Tremendous improvement in GF has been observed which improved from single-digit to several hundreds. The highest GF obtained so far is ~255, showing 75 times improvement compared with the ones without the SCIM implementation. The crack initiation strain (CIS) is characterized by sonication and centrifugation time. It is determined experimentally that the maximum CIS of 3.5% can be achieved with sonication time of 40 min and increasing centrifuge time has an in-significantly dropping effect on CIS. Excellent stability/reproducibility has been proved/demonstrated on SCIM implemented sensors through a rigorous 12,500 tensile cycle test on DMA. The performance of sensor is practically demonstrated in tension and bending on glass fiber reinforced polymer (GFRP) structures.

## Introduction

With both theoretical and experimental evidences, carbon nanotubes (CNTs) inherit excellent mechanical strength, Young’s modulus, electrical and thermal conductivities of approximately 100 GPa^1,2^, 1 TPa^3,4^, 3 × 10^4^ S/cm^5,6^ and 2000–3500 W/m·K^7,8^ respectively. With the finding of piezoresistive properties of CNTs, having a maximum reported gauge factor (GF) of up to 2900^9,10^, great interest is shown in exploring it for the development of smart and multifunctional sensors for structural health monitoring (SHM) applications over the past few decades^9–12^. Assembling CNTs to form a large size bulk structure is important for practical applications, e.g., development of SHM sensors, energy storing devices, display devices, and structural integrity applications. Buckypaper has the principal advantage over other forms of CNT structures such as CNT fibers with limited one direction monitoring^13,14^ and CNT foams with bulky dimensions^15,16^. In terms of piezoresistive/electrical/mechanical properties, CNTs based structures are lagging behind the individual properties of CNTs. Extensive research has still been conducted to improve the properties of buckypaper, as the superior properties of individual CNT deteriorate significantly when attempted to combine them to form buckypaper for practical applications^17^. The property deterioration is generally attributed to two causes, one is the random arrangement of the uneven CNT network in the buckypaper^18–20^ because of strong Van der Waal’s attractions, resulting in entanglement and bundling, and the second is the addition of impurities during the synthesis of buckypaper, in particular, the addition of surfactants in dispersion^21^.

The primary performance parameter for the adaptability of strain sensors in the SHM field is based on their large working strain range and their sensitivity to strain, i.e., a large variation in electrical resistance with less change in strain leads to a very sensitive sensor^22,23^. Sensitivity can be characterized as GF, which can be defined as fractional electrical resistance divided by deformation. Mathematically, GF can be computed as GF = (dR/R)/ε. where dR is the change in resistance, R is the initial resistance and ε is the mechanical strain. Performance degradations of CNT in the format of buckypaper are reflected by small strain magnitudes ranging between 0.2% to 1% strain^21,24,25^ and small GF ranging between 0.38 to 1.7^22^.

The process for the synthesis of the buckypaper is carried out by assembling the solution phase, in which the essential step is to prepare a well-homogenized CNT dispersion solution and convert it into a buckypaper through hydroentanglement^26^, hydraulic press^21^ and vacuum filtration^27–33^. All of these methods have two things in common: the random arrangement of the CNTs in the bulk form and the significant deterioration of the properties.

To investigate the cause of the properties deterioration and to improve them while maintaining the purity of CNTs, extensive studies have been carried out to study the microstructure of the CNTs and establish a relationship between the parameters of the CNTs and its piezoresistive behavior, electrical and mechanical properties^19,34–38^. From these investigations, it can be concluded that the reported properties of the CNT in the bulk form are lagging behind the extraordinary properties of individual CNTs. A well-ordered and covalently linked CNT network is crucial for exceptional properties in the bulk form, but it is very difficult to obtain a well-organized CNT network due to the likelihood of entanglement and bundling during synthesize of buckypaper^17,27^.

To improve the piezoresistive properties in terms of GF and high workable strain regions and to establish a relationship between microstructural parameters and the macro-level properties, various techniques have been recently established, most of which compromise the purity of the CNT network, which uses epoxides or other nanomaterials with CNT to synthesize a well-ordered CNT network. For instance, Luo, S. *et al*. established a relationship between the GF of the sensor and the dimensions of CNT and recorded a GF of 5 for large strains^39^. Shaokai, W. *et al*. compared the in-line properties of wet-stretching CNT film with dry-stretching and reported a stable piezoresistive behavior with the GF factor of 13.2^40^. Ying, Y. *et al*. introduces the WASS (Water-Assisted Shear Stretching) approach, which can significantly improve the alignment of CNTs. The recorded workable strain region was 8.07%^41^. George, T. *et al*. reported a workable strain region of 2.35% in their efforts to improve the performance of buckypaper by epoxidation of CNTs^42^. Xin, W. *et al*. used CNT/nylon composites, indicating that the workable strain region decreases from 2% to 1% as pre-stretching increases from 0% to 7%^43^.

**Figure 1:**
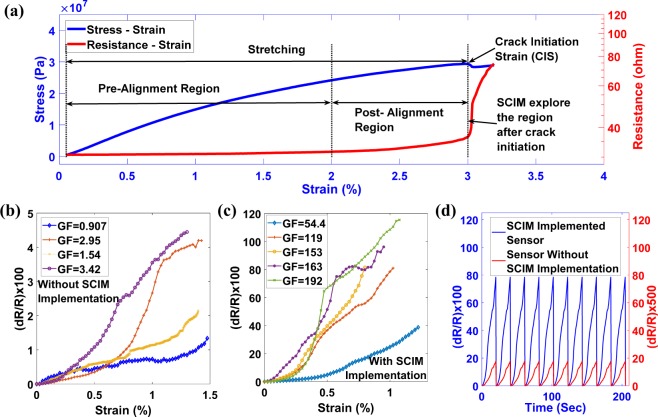
(**a**) Stress & resistance change behavior of buckypaper based sensor under tensile stretching. The region after crack initiation is much superior in terms of sensor sensitivity, which is explored in the current study of establishing SCIM. (**b**,**c**) Resistance change with a corresponding strain change without and with the SCIM implementation. All sensors were subjected to tensile strain cycles at a rate of 3%/min and were strained between the corresponding LSR and the USR. By implementing SCIM, the improvement in the GF was tremendous from single digit to several tens and hundreds. (**d**) Resistance change pattern of 10 cycles for sensors with and without SCIM implementation.

While the mechanical and electrical properties of CNT buckypaper have been extensively studied to register improvements through the specific use of stretching techniques by aligning the CNT random network, the improvement of piezoresistive properties, particularly the GF, does not adequately achieved and no one ever achieve high GF of reproducible buckypaper based strain sensor in the magnitude of 3 digits. In this study, a novel SCIM protocol is developed to improve sensor performance for high GF by generating microcracks in hybrid PI-enabled buckypaper sensors. As a substrate of the sensor, a PI tape was used, which improved the failure strain of the sensor and prevented sudden destruction of the sensor after the initiation of the crack. In SCIM, the technique of stretching the buckypaper sensor has adopted to intentionally create micro-cracks in the sensor to utilize the superior sensing characteristics due to the presence of a crack. Previous studies of stretching the buckypaper based sensor employed only to align the CNTs in buckypaper. The strain at which the crack initiated was regarded as the failure strain of the sensor. The SCIM considers the failure strain of previous studies as CIS. Figure 1(a) distinguishes the SCIM from previous work of stretching, utilized for alignment of CNTs in buckypaper, through stress & resistance change behavior with a change of strain in the sensor. The microcracks in the SCIM implemented buckypaper based sensor were generated by stretching the sensor in dynamic mechanical analyzer (DMA), and crack geometries were analyzed with a scanning electron microscope (SEM) and through an optical microscope. A series of experiments suggested a very strong and distinct relationship between piezoresistive properties, crack geometry and dispersion processing parameters. It has been found that GF depends mainly on the crack opening and the crack initiation strain (CIS), which is the maximum allowable working strain for the sensor, depends mainly on the dispersion preprocessing parameters, especially the sonication time. Accordingly, in this study, the GF was characterized by crack opening and the CIS was characterized with sonication and centrifugation time. It was found that the GF increased non-linearly from ~20 to ~255 when the crack opening increased from 0.5 μm to 8 μm. The mechanism that causes the increase in GF with the increase in the crack opening is based on the basic principle of electrical discontinuity, i.e., a larger crack width results in a significant electrical discontinuity in the sensor, which allows a enormous change in the electrical resistance and, therefore, a larger GF. It was found that the CIS increased from 2.8% to 3.5% when the sonication time increased from 20 minutes to 40 minutes. The reason for the improvement of CIS has been studied with SEM, which suggest that cracks have been generated at the boundary of the higher magnitude of CNT aggregation in the buckypaper. With the increase in sonication time, a well-homogenized dispersion mixture with a lower aggregation of the CNTs can be obtained. Therefore, the crack appears at higher strain levels with increasing sonication time. As the sonication time increased above 40 minutes, the CIS decreased dramatically to 1.8%, which was further reduced to 1.6% when the centrifugation time was increased. This behavior suggests that individual CNTs disintegrate into smaller size particles above 40 minutes of sonication time, thus increasing the aggregation of CNT in the dispersion due to smaller size particles, resulting in a lower level of CIS. Similarly, as the centrifugation time increases, the large size particles (removal of which increases CNT aggregation in dispersion and thus decreases the CIS) and heavy particles (removal of which decreases CNT aggregation in dispersion and thus increases the CIS) were removed from the dispersion, so the centrifugation time has marginally reducing impact on the CIS. Comparable behavior of decreasing working strain region with increasing mechanical and electrical properties was observed when the random network of CNTs was aligned using stretching techniques^44^. The two parameters of the sensor, i.e., the GF and the CIS, can be controlled independently, allowing people to easily reach the high GF and high working strain level for the sensor.

This study provides an SHM buckypaper based sensor that not only saves energy in SHM systems through improved electrical conductivity, but also provides a highly sensitive sensor with reasonably enhanced working strain in the linear region both in static and dynamic frequency environments, where a high sensitivity of the sensors is desired. To demonstrate the actual applications of hybrid sensors with significantly improved properties, the SCIM implemented sensor performance has been successfully demonstrated on the specimen of glass fiber reinforced plastic (GFRP) in tension and bending directions, showing ~25 and ~12 times superior sensor sensitivity in tension and bending respectively.

## Results and Discussion

### SCIM implementation

To efficiently generate micro-crack for enhancing the GF of PI-tape enabled buckypaper based sensor, the SCIM protocol is developed through which the GF of the sensor is significantly improved from single-digit to several tens and thousands which can be seen in the results of Fig. 1. A series of experiments were designed by varying the centrifugation time to observe the sensitivity of the sensor. It was observed that without the implementation of SCIM, the maximum GF of the sensor was ~3.42. Subject to the implementation of SCIM, the sensor GF was improved to reach the range of ~150 to ~250, as shown in Fig. 1(c). To compare the results of the change in resistance with and without SCIM implementation, 10 tensile strain cycles are shown in Fig. 1(d), which demonstrates the significance of SCIM implementation to improve the piezoresistive properties of Sensors.

### SCIM foundation

The foundation of SCIM is based on the fact that the generated micro-cracks constitute an electrical discontinuity in the sensor and when the sensor is stretched, the discontinuity is elongated. Due to the increase in discontinuity, the variation of the resistance of the sensor increases, which improves the GF. Alternatively, a large crack opening can degrade the life of the sensor, which can lead to sensor failure due to crack propagation. On the one hand, the sensitivity of the sensor can increase with increasing crack opening; on the other hand, the stability of the sensor can impair. It has therefore been deemed necessary to study the conditions as SCIM protocol that not only improves the sensitivity but does not affect the stability of the sensor either.

**Figure 2:**
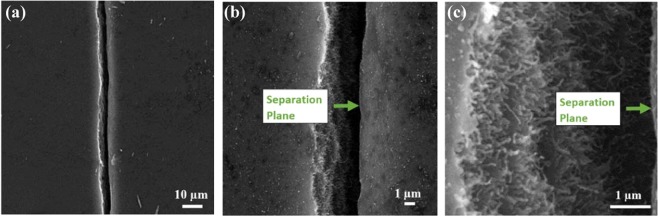
(**a–c**) SEM images of the representative cracked region having a magnification of (**a**) 1,000, (**b**) 5,000 and (**c**) 20,000 respectively, showing only one primary crack generated on the sensor indicating its weakest part.

First, efforts were made to develop sensors with similar sensing performance. Although great care has been taken to synthesize buckypaper sensors with a similar dispersion process and crack initiation parameters, it has been found that after the implementation of the SCIM, the sensor performance in relation to GF and the working strain region varied from one sensor to another. This is probably due to the variation of crack geometry, generated uncontrollably when stretched in the DMA due to the non-uniform aggregation present in the random arrangement of the CNTs in buckypaper. From SEM the presumption was confirmed that the implementation of SCIM in sensors, synthesized under similar processing conditions, has different crack openings, which resulted in different sensing performance. The SEM examination of the crack geometry shown in Fig. 2 also shows that cracks were generated in the most vulnerable area; certainly, this is the area where there are significant misalignment and aggregation of CNTs.

### Characterization of working strain and GF

So far, it has been confirmed that the generation of microcracks in the sensor can improve sensor performance in terms of the GF and working strain region, but the problem of characterizing sensor performance in specific quantifiable parameters still exists. The length of the CNTs in dispersion for synthesizing buckypaper can be considered as a vital factor in characterizing sensor performance. The length and aggregation of CNTs in a random CNT network can be adjusted as a function of sonication time and centrifugation time. The presumption was that prolonging the sonication time can reduce the CNT length and prolonging the centrifugation time can reduce the magnitude of heavy particles from the dispersion, which could affect the geometry of the crack and, therefore, the piezoresistive properties of the sensors.

**Figure 3:**
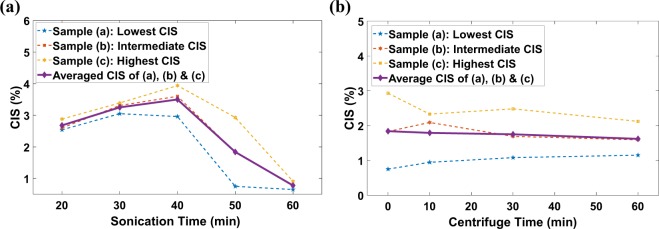
(**a**) Effect of sonication time on the average CIS (working strain region). After 40 minutes of effective sonication time, the length of CNTs decreased due to which cracks were generated at much lower strain levels. (**b**) Effect of centrifuge time on CIS. A slight downward trend suggests the removal of larger CNTs to reduce CIS.

Based on this hypothesis, we first examined the effect of sonication time; all preprocessing parameters mentioned in the “Method” section remained the same for the synthesis of buckypaper. The sonication time was increased from 20 minutes to 60 minutes in 10 minutes increment. According to the preparation and implementation steps of SCIM mentioned in the “Method” section, the sensors were developed, and performance was evaluated. It has been observed from experimental results, as shown in Fig. 3(a), that the working strain region in terms of CIS increases with the sonication time up to 40 minutes. The extension of the sonication time improves the homogenization of the dispersion, which reduces the magnitude of aggregation in the CNT network and, therefore, increases the magnitude of the CIS. After 40 minutes of sonication, the CIS decreases rapidly, as sonication time exceeding 40 minutes would shorten the length of the CNT, which would increase the degree of aggregation in the CNT network. Therefore, the crack was generated in the lower strain region when the sonication time exceeded 40 minutes.

Second, the effect of centrifugation time on the working strain region in terms of CIS was examined. The sensors were developed with buckypaper synthesized for an effective sonication time of 50 minutes to further investigate the behavior after CNT breakage due to excessive sonication. All other preprocessing parameters for the buckypaper synthesis mentioned in the “Method” section remained the same. The centrifugation time was increased from 0 minutes to 60 minutes with an increment of 10 minutes. The developed sensors were stretched in DMA to study the effect of centrifugation time on the CIS. From the experimental results, as shown in Fig. 3(b), it was observed that the CIS decreases insignificantly with increasing centrifugation time. The behavior was such because the large particles, which prevent CNT aggregation, removed from the dispersion as a result of an increase in the centrifugation time.

Fortunately, a strong and obvious relationship was observed between the working strain region and the dispersion parameters, i.e., the sonication and centrifugation time, to allow users to synthesize sensors with an adjustable working strain region by changing the sonication and centrifugation time as shown in Fig. 3. On the contrary, the GF still needed to be characterized by a quantifiable parameter as no relationship was observed between the GF and the sonication/centrifugation time due to the fact that the micro-cracks generated in the sensor through the tensile stretching in DMA was not controllable, i.e., cracks were generated at different CIS levels and of different crack geometry specifically the crack width.

**Figure 4:**
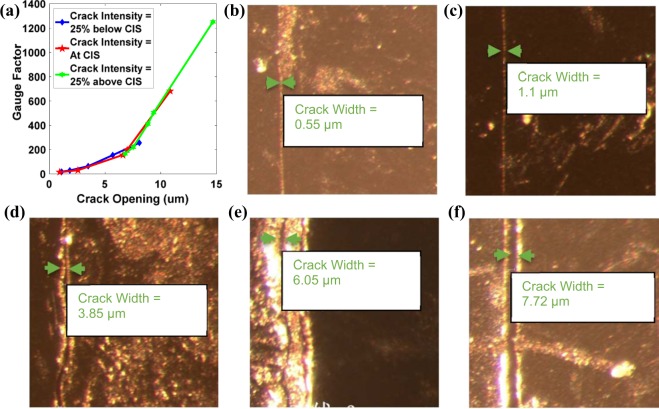
(**a**) Characterization of sensor gauge factor with the crack opening. (**b**–**f**) Optical microscope images of crack width measurement of five samples at the crack intensity of 25% below the CIS level in increasing order.

An attempt was made to characterize the GF of the SCIM implemented sensor with corresponding crack width for three crack intensity levels, which were 25% below CIS, at CIS and 25% above CIS levels. Fortunately, this time, a very strong relationship between GF and the crack width was observed. It has been observed that the GF increases non-linearly with increasing crack width, as shown in Fig. 4(a). Figure 4(b–f) shows optical microscope images (using which the crack width was measured) of 5 samples with crack intensity less than 25% CIS. The lowest GF obtained by the SCIM implemented sensor was ~25 with a crack width of ~0.55 μm. The maximum GF of ~1200 was obtained when the crack width was ~15 μm, but the sensor was unstable, i.e., failure of the sensor occurred after a few tensile strain cycles, which was unacceptable.

After characterizing the two main parameters of sensor performance, namely GF and the working strain region with crack geometry and preprocessing dispersion parameters respectively, the biggest concern was the stability of the sensor, as most of the SCIM implemented sensors failed after few tensile cycles at a crack intensity of above 25% CIS. Therefore, a criterion had to be set to obtain a stable sensor which performs its function without failure. During the characterization of the GF, it was observed that the sensors could work with stability, i.e., without crack propagation, if it possesses a crack having width <8 μm. Subject to crack intensity levels of “25% above CIS” and “at CIS”, it was found very difficult to obtain a sensor having a crack width <8 μm. Therefore, it was concluded that these crack intensity levels result in an unstable crack and so thus an unstable sensor. As a result of this key conclusion, we can say that SCIM implemented sensor having crack width below 8 µm and operating under crack intensity “25% below CIS levels” would function with stability and as shown in Fig. 4(a) possess a GF of ~255.

### Stability of sensor

**Figure 5:**
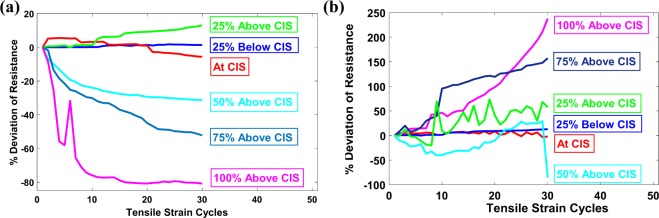
(**a**) Displays cycle to cycle deviation of sensor resistance at LSR. The sensor remains stable at the LSR when the USR remains 25% below the CIS. (**b**) Displays cycle to cycle deviation of sensor resistance at USR. The sensor remains stable at the USR when the USR remains at 25% below the CIS.

To justify the assertion concerning the stability of the sensor, a series of tests were carried out. The stability of the sensor performance depends entirely on the stability of the generated micro-cracks and was evaluated by the resistance-strain curve during the tensile testing cycles. The sensor can be considered as stable if it provides reproducible resistance cycles against repeating tensile cycles. The stability of the crack in the sensor was studied for 6 crack intensity levels, for which the upper strain region/limit (USR) during tensile strain cycles were kept at 25% below CIS, at CIS, 25% above CIS, 50% above CIS, 75% above CIS and 100% above CIS. The sensors were subjected to tensile cycles in the DMA at a strain rate of 3%/min. Experimental observations have shown that the deviation between the upper and lower resistance regions was negligible when the USR was kept at the level of 25% below CIS i.e., there was no evidence of crack propagation when the sensors cycled between lower strain region/limit (LSR) and 25% below CIS levels. It can be seen in Fig. 5(a,b) that the deviation of the resistance varied from one cycle to another when the sensors were subjected to tensile cycles higher than the CIS levels. Therefore, in the presented SCIM, it was recommended to maintain the USR at 25% below the CIS level, so that the sensor operates consistently without crack propagation.

**Figure 6:**
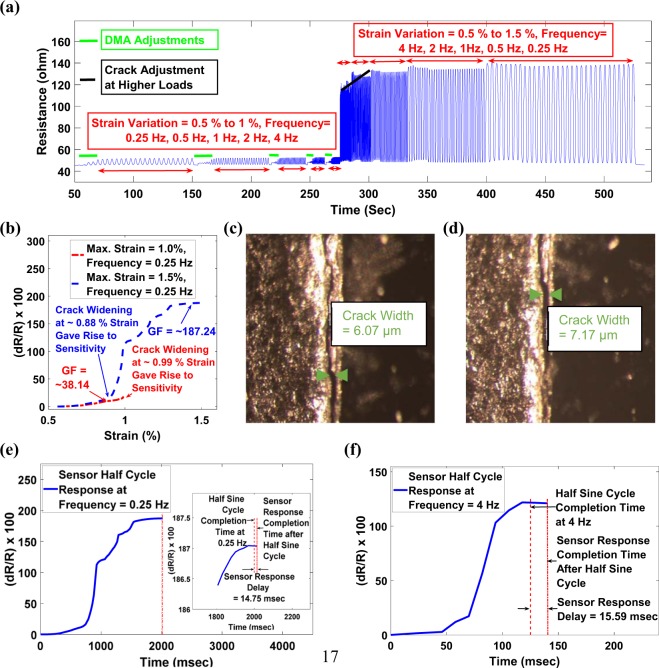
(**a**) Response of SCIM implemented sensor under dynamic tensile testing conditions through variation of strain and frequencies. (**b**) Sensitivity comparison between first loading cycle set i.e., Max. Strain = 1%, Frequency = 0.25 Hz and last loading cycle set i.e. Max. Strain = 1.5%, Frequency = 0.25 Hz. The crack is adjusted during high strain cycles resulting in a crack widening effect taking place at ~0.88% strain for last cycles compared to crack widening effect taking place at ~0.99% strain for initial cycles. (**c**,**d**) Crack width analysis before and after cyclic tensile tests with multi-loads & multi-frequencies. (**e,f**) Sensor response time analysis under a dynamic environment at 0.25 Hz and 4 Hz respectively showing a delay of 14.76 msec and 15.59 msec respectively at 0.25 Hz and 4 Hz frequencies.

In addition, the stability of the SCIM implemented sensor was judged in a dynamic environment through multi-load and multi-frequency tensile cyclic loadings in DMA. The SCIM dominated regions i.e., the effect of crack widening on superior sensor characteristics (GF), in this representative sensor, was dominant above ~0.5% strains; therefore, the load cases were designed accordingly. The sensor was subjected to 0.5% to 1% strain and 0.5% to 1.5% strain under a series of low and high frequencies, i.e., 0.25 Hz, 0.5 Hz, 1 Hz, 2 Hz, and 4 Hz. As shown in Fig. 6(a,b), sensor sensitivity increased exponentially when subjected to higher strains due to the crack widening effect. It can also be seen in Fig. 6(a) that when the sensor was subjected to a higher strain load case i.e., upto 1.5% strain, there was a slight increase in upper bound resistance at the initial cycles, which was stabilized after few cycles. Also, when the sensor initial load case i.e., 1% strain, 0.25 Hz frequency, was compared with last load case i.e., 1.5% strain, 0.25 Hz frequency, as shown in Fig. 6(b), the crack widening effect was witnessed comparatively at the marginally smaller magnitude of strain in the last load case. This shift in crack widening effect on sensor sensitivity along with an increase in upper bound resistance at higher magnitude loadings suggest that the crack in the sensor adjusted during the dynamic loading environment. The morphology of crack opening in terms of crack width was further analyzed before and after dynamic testing using the optical microscope as cracks generated in the sensor through SCIM implementation were of micron size, which can easily be analyzed using the available magnification of the optical microscope. Figure 6(c,d) confirms that crack width was adjusted and marginally increased after multi-load and multi-frequency tensile cyclic loadings but remained within the stable region of SCIM i.e., <8 µm. The maximum GF achieved for this representative sensor was found to be ~187, which was in accordance with the results of GF characterization with crack width, as shown in Fig. 4(a), indicating high reproducibility of the sensor in terms of piezoresistive sensing characteristics using SCIM protocols.

The sensor response time is very important in practical applications for SHM sensors. Therefore, it is necessary to analyze the sensor response in a dynamic environment. The sensor response time was computed at low and high frequencies i.e., at 0.25 Hz and 4 Hz, by plotting the respective cyclic resistance change response with time. The time taken by the sensor to complete the cycle was judged by comparing the resistance change of sensor with the respective ideal time of cycle completion at 0.25 Hz and 4 Hz frequencies. As shown in Fig. 6(e,f), the delay in sensor response at 0.25 Hz and 4 Hz frequencies was found to be 14.75 msec and 15.59 msec respectively. The delay in the response time of sensor are much superior than mentioned in recent research where the sensor response time was estimated to be 87 msec^45^ for MWCNT based sensor.

It can be concluded from the results of multi-load and multi-frequency tensile cyclic loading tests that the sensor performance under the dynamic environment is stable i.e., there are no signs of crack propagation. The results confirms the applicability of SCIM implemented sensor in a dynamic environment.

### Survivability of sensor

**Figure 7:**
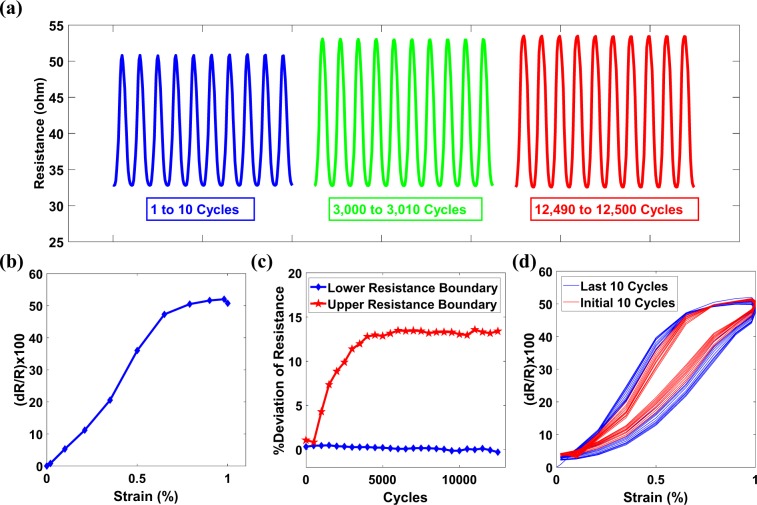
(**a**) Durability test of 12,500 tensile sine cycles showing stable and repeatable resistance from 3,000 cycles to 12,500 cycles. (**b**) SCIM implemented sensor sensitivity during the durability test. (**c**) Cycle-to-Cycle deviation of upper and lower resistance boundaries measured from initial upper and lower resistances. An insignificant deviation can be observed both in the lower resistance boundary and in the upper resistance boundary (after 3000 cycles). (**d**) The hysteresis shows that the initial and final points of the cycles remained the same throughout the cycles, but there was a slight change in resistance between the initial and final points. In general, it can be concluded from the hysteresis that the sensor has remained stable during the durability test.

The durability of the CNTs based sensor in terms of its survivability is judged in recent studies by subjecting the sensor under tensile loadings for elongated duration from 3,000 cycles^46^ to 10,000^13,47,48^. In this study, the durability of SCIM implemented sensor is judged by subjecting the sensor to rigorous 12,500 sinusoidal tensile cycles in DMA. The amplitude of the sine wave was kept constant during the cycles at 0.5% strain. Overall, the sensor was exposed to a strain of 1% with a sine frequency of 0.5 Hz. During the sine test, the crack in the sensor was first adjusted itself and the upper boundary of sensor resistance was slightly increased up to 3,000 cycles. The deviation of resistance at the upper boundary from the first cycle to 3,000 cycles was measured to be ~13%. Thereafter, the crack remained stable for the next 9,500 cycles, with the upper and lower resistance limits remained at the same level as shown in Fig. 7(a). It was observed that the sensor resistance response follows the sine wave having GF magnitude of ~83 as shown in Fig. 7(b). To verify that the sensor remained stable throughout sine cycles, the sensor performance was analyzed in terms of the cycle-to-cycle deviation of upper and lower resistance boundaries from the initial cycle as shown in Fig. 7(c) and through resistance-strain hysteresis as shown in Fig. 7(d). By analyzing the resistance deviation, it was observed that for the initial cycles, the lower resistance boundary has an insignificant deviation and that the upper resistance boundary experience a marginal deviation of up to 3,000 cycles, after which the upper resistance boundary stabilized and remained steady until the end of the test at 12,500 cycles.

### Practical application demonstration

**Figure 8:**
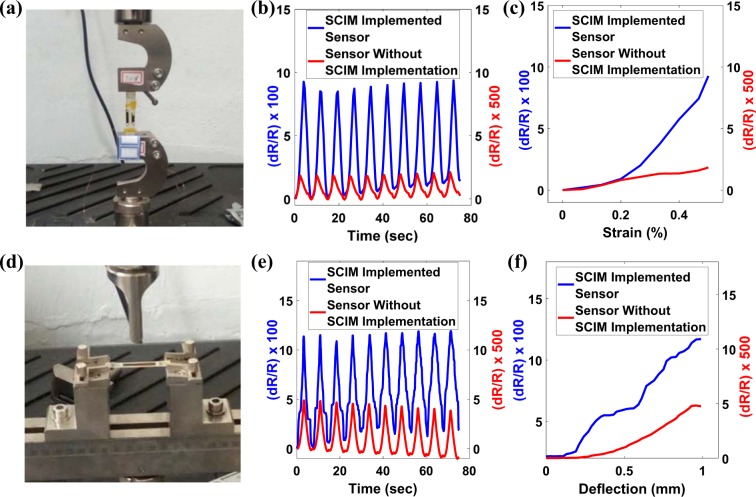
(**a,d**) Machine setup of tensile & 3-point bending tests respectively. (**b,e**) Comparison of 10 sensor performance cycles with and without SCIM implementation for tensile and 3-point bending tests respectively. (**c,f**) Comparison of sensor sensitivity with and without SCIM implementation for tensile and 3-point bending tests respectively. The results shown in (**b,c,e,f**) prove that sensors with SCIM implementation outperform the sensors without SCIM implementation in terms of sensor sensitivity both in tensile and bending loading cases.

To demonstrate the practicality of the sensor and to compare the performance of the sensor with and without SCIM implementation, the sensors were fabricated using a single piece of buckypaper and tested on GFRP samples using the universal tensile testing setup in MTS under tensile and bending loading conditions. The GFRP laminate was produced by a vacuum-assisted prepreg curing process as introduced previously^49,50^. A GFRP specimen of rectangular dimensions 10 mm wide and 50 mm length was cut from a cured GFRP sheet. Two sensors, one with the SCIM implementation and the other without, were attached side by side in the GFRP specimen. The setup for tensile and 3-point bending test is shown in Fig. 8(a,d), respectively. Tensile and bending cyclic tests were performed between the strain of 0% to 0.5% at a frequency of 8 cycles/min and a bending deflection of 0 mm to 1 mm at a rate of 16 mm / min respectively. It has been discovered that both sensors operate with stability. However, the sensitivity of the sensor with the SCIM implementation outperforms the counterpart, as shown in Fig. 8(b,c,e,f) for tensile and bending tests respectively.

## Conclusion

The SCIM for developing a PI-Tape enabled MWCNT buckypaper based sensor with improved piezoresistive properties, particularly GF, has been successfully introduced. With this method, the GF of buckypaper sensors can be enhanced from several tens to hundreds by deliberately creating stable cracks in the buckypaper sensor. The methodology to generate stable cracks has been studied and it was found that micro-cracks will remain stable if it has an opening below 8 µm and when the sensor operated below 25% of the CIS level. It was observed that GF of SCIM implemented sensor entirely depends upon the crack geometry and although the sensors developed and evolved under similar processing and testing conditions, the crack geometry varies from sensor to sensor, so thus the sensing characteristics in terms of GF. This finding suggests that the cracks generated by DMA are uncontrollable. Therefore, it is necessary to develop a mechanism of crack initiation, which can generate identical cracks so that the sensors produced under similar processing conditions and subjected to similar testing conditions, have the same sensing characteristics. The GF was characterized with crack width and it was found that the maximum GF can reach the magnitude of ~255. The CIS was characterized by sonication and centrifugation time. It was found that the CIS decreased dramatically as sonication time increased above 40 minutes due to breakage of CNTs and with increasing centrifugation time, the CIS marginally decreased. It is established through series of experimentations that PI-tape enabled buckypaper sensor with the SCIM implementation can work with stability and much-improved sensitivity which can lead towards the implementation of SCIM sensors in industrial application especially in space and aerospace industry where higher sensitivity of sensors can improve their design and structure health monitoring throughout their mission and operational life.

## Methods

### Materials and equipment

Multi-wall carbon nanotubes (MWCNT) having an external diameter of 4 nm to 6 nm, a length of 10 μm to 20 μm with a purity specified by the supplier of 98% were obtained from Time Nano China Inc. The commercially available PI tape was used, having a thickness of 40 μm. Triton X-100 surfactant was purchased from Sigma Aldrich. To prepare the dispersion, an ultrasonic sonicator was used with the power of 480 W. To separate large CNTs, the Eppendorf 5418 centrifuge at 5,000 rpm was used. Millipore PVDF (polyvinylidene fluoride) membranes were used to filter the MWCNT in a filtration set up with a vacuum pressure of 0.95 bar. With the Universal Laser System DLS 2.3, the buckypaper was cut into a rectangular shaped sensor. The thickness of the buckypaper and the width of cracks in the sensors were measured using a Nikon microscope. The Discovery DMA 850 (TA Instruments Inc.) was used to intentionally create stable cracks in the buckypaper sensor and to characterize the sensor performance. The Keithley DMM7510 digital multimeter was used to measure resistance and LabVIEW for data acquisition during the sensor tensile tests. MTS was used for tensile and bending applications on GFRP composite.

### Optimization of dispersion parameters

Preprocessing parameters of dispersion such as the amount of CNTs, de-ionized (DI) water, surfactant, sonication, and centrifugation time as well as the material and the pore size of filtration membrane influence the properties of the buckypaper sensor. Many researchers have used different combinations of preprocessing dispersion parameters for their applications^27–32^. In this study, we optimized the preprocessing parameters for minimum resistivity through a series of optimization experiments, which are summarized in supporting material. It concludes that the resistivity can reach a minimum of 0.1234 ohm-mm (conductivity = 8104 S/m) when the volume of 70 ml, MWCNT = 90 mg mixed with DI water = 100 ml and surfactant (TX-100) = 1.5 ml, sonicated for 30 minutes and centrifuged for 30 minutes, was filtered with a 0.22 μm PVDF membrane. The conductivity obtained was comparatively higher than reported in recent studies ranging between 3620 S/m and 6640 S/m^30,33,51^ for pristine MWCNT buckypapers. In addition to the advantage of approaching the exceptional properties of CNTs, improving the conductivity by optimizing the processing parameters provides an additional energy-saving feature due to the improved conductivity of the sensor. The GF and workable strain region of pristine buckypaper were improved by optimizing the preprocessing parameters to 3.42 and 1.25%, respectively.

### Synthesis of buckypaper

Buckypaper was synthesized using the filtration method with optimized preprocessing dispersion parameters for minimal resistivity. In the vacuum filtration process, a homogenized dispersion of CNT was filtered using a filtration membrane in a filtration set up with a vacuum pressure of 0.95 bar. After filtration, the buckypaper on the filtration membrane was immersed in DI water for several minutes to remove the surfactant from the buckypaper. After drying, the buckypaper was easily peeled off from the filtration membrane.

### Preparation and installation of buckypaper sensor on DMA

**Figure 9:**
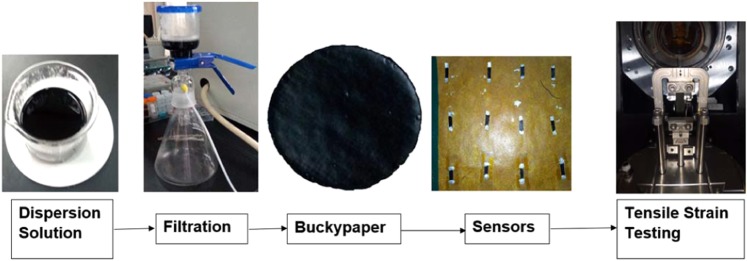
Schematic block diagram of the flowchart illustrating the overall methodology from dispersion preparation to tensile test.

The synthesized buckypaper was cut into rectangular shape and was placed over the PI tape and pressed gently using the thumb to ensure that the entire surface of buckypaper is bonded evenly on the PI tape. Then the PI tape was cut to take the dimensions of rectangular buckypaper. In this configuration, the top surface of the PI-tape enabled buckypaper sensor is visualized as buckypaper and the bottom surface is visualized as semi-transparent PI-tape. The interaction between PI-tape and buckypaper is guaranteed by the existed adhesive layer on top of PI-tape. For clarity, the step by step sequence, in terms of a pictorial block diagram, of sensor preparation is added in Supporting Material Fig. S7. The PI-tape serves the purpose to intact the buckypaper i.e., to avoid sudden failure of buckypaper sensor after the crack initiation. To minimize the contact resistance between sensor and DMA clamps, the contacting areas of sensors with DMA clamps were applied with commercially available silver paint. To check and record the resistance during operation, the DMA clamps were connected to the Keithley digital multimeter with copper wires. The broad level diagram of the process flow from the preparation of the dispersion for the synthesis of the buckypaper to the characterization tensile tests of the sensor is shown in Fig. 9, and the detailed illustration of the step-by-step sequence of key activities of process flow is included in the Supporting Material Fig. S8.

### SCIM-method of sensor performance enhancement

The performance of the sensor in terms of GF can be significantly improved by the formation of microcracks in the sensor. In the current study of the novel SCIM, the micro-cracks were generated by stretching the sensor in DMA. As microstructure/CNT aggregation in buckypaper vary one location to another and level/magnitude of aggregation varies sensor to sensor. Therefore, the cracks have been generated at different strain magnitudes and of different crack geometry (width). A strong correlation between GF and crack width was established by a series of experiments that varied the intensity of the crack. It was found that the sensor would function without failure if the USR will be fixed below 25% of the CIS level and if crack width is below 8 µm. The sensors are required to perform under tension cycles for which it is necessary to identify the stable region of strain where the crack would not propagate. To determine the stable strain region, it is necessary to determine the LSR and the USR, both related to CIS. The step-by-step process flow of SCIM is described below as a representative example for a better understanding of the method.

### Investigating lower strain region (LSR)

**Figure 10:**
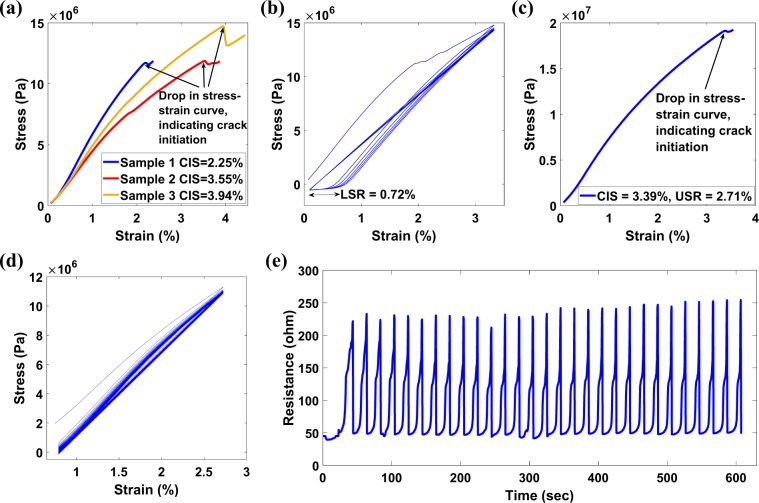
(**a**) CIS of three buckypaper-based representative sensors. (**b**) Stress-strain diagram of a representative sensor for investigating the amount of permanent deformation (LSR) after six tensile strain cycles. (**c**) Crack initiation cycle of a representative sensor in which USR is calculated by reducing the CIS by 25%. (**d**) Stress-strain diagram for the tensile strain cycles performed between LSR and USR, i.e., between 0.72% strain to 2.71% strain at a strain rate of 3%/min. (**e**) Resistance changes in the sensor during tensile cycles. The calculated GF for this representative sensor is ~190.

It was observed that the PI-enabled buckypaper based sensor undergoes plastic deformation during the initial few tensile cycles. The plastic deformation of the sensor is termed as strain hardening in SCIM. The LSR is the zero-strain level obtained after the strain hardening, i.e., the value of strain where stress is equal to zero after strain hardening. The LSR must be calculated to ensure consistent sensor performance. For investigating the LSR, the CIS of three representative sensors was investigated and averaged, as shown in Fig. 10(a). The initiation of crack was indicated by a drop in the stress-strain curve or by a rapid increase in resistance. In these three representative sensors, the cracks have been generated at 2.25%, 3.55%, and 3.94% strain. The average CIS of these three representative sensors has been computed to be 3.25% strain. To investigate the LSR, another representative sensor was undergone six tensile strain cycles at the rate of 1.8%/minute between 0.05% strain to average CIS (in this representative case between 0.05% strain to 3.25% strain), which can be seen in Fig. 10(b). At the end of the sixth cycle, the strain level of the sensor at which corresponding stress was equal to zero i.e., the LSR has been noted through the stress-strain diagram. In this representative case, the LSR has been calculated to be 0.72% strain. During the computation of sensor performance parameters i.e., the GF and working strain region, the LSR has been subtracted from the USR to get actual strain region of the sensor.

### Creation of crack and calculating upper strain region (USR)

After determining the LSR using representative sensors, the USR of the sensor can directly be computed through CIS for each sensor whose performance is required to be investigated. The sensor was stretched at a constant strain rate of 1.8%/minute until the crack was generated, which was identified by a slight drop in the stress-strain curve shown in Fig. 10(c). The USR was calculated by 25% reduction of CIS (in this representative sensor, the USR was found to 2.71%). The 25% reduction in CIS for computation of USR is the experimentally determined value, found through several iterations, to keep the crack stable in the sensor.

### Cyclic tensile testing of sensor

After finding LSR and USR, the sensor was subjected to cyclic loading and unloading between the LSR and the USR strain range. The rate of strain cycles was 3%/min. The stress-strain curve and the corresponding resistance-time diagram of the representative sensor are shown in Fig. 10(d,e) respectively, in which the consistent performance of the sensor is evident. It can be seen in Fig. 10(e) that the sensor resistance change was from ~50 ohms to ~240 ohms for a total strain change of ~2%. Therefore, the GF of the sensor can be calculated and determined for this representative sample as ~190, which is much higher than the GF of the sensor without the implementation of the SCIM.

## Supplementary information

Supplementary Information
