# Supplementary material for: Micro-Crack Induced Buckypaper/PI Tape Hybrid Sensors with Enhanced and Tunable Piezo-Resistive Properties
Source: Sci Rep. 2019 Nov 15;9:16920. doi: 10.1038/s41598-019-53222-1 (PMC6858318; doi:10.1038/s41598-019-53222-1)
Supplement: Supplementary file 1 — Supplementary Information [file 41598_2019_53222_MOESM1_ESM.docx]

Supporting Information

**Micro-Crack Induced Buckypaper/PI Tape Hybrid Sensors with Enhanced and Tunable Piezo-Resistive Properties**

Mustafa Danish^1^, Sida Luo^1,^*

^1^Beihang University, School of Mechanical Engineering & Automation, Beijing, 100191, China

*s.luo@buaa.edu.cn

- **Optimization of Pre-Processing Dispersion Parameters for Minimum Resistivity of MWCNT Buckypaper**

Series of experiments were designed to optimize the dispersion preprocessing parameters to achieve a minimum resistivity of the MWCNT buckypaper. The parameters studied include the amount of CNT, deionized water, surfactant, sonication and centrifugation time, material and pore size of the filtration membrane. To optimize these parameters for minimum resistivity, all preprocessing parameters except for the optimized one have been retained. For each buckypaper, the resistivity of the three samples was calculated and averaged to avoid / minimize human errors. The entire optimization process is graphically summarized in Figure S1-S6 and in Table S1.

A number of experimental studies have shown that a minimum resistivity of 0.1234 ohm-mm (conductivity = 8104 S / m) can be achieved by mixing a volume of 70 ml of good quality MWCNT = 90 mg mixed with DI water = 100 ml and surfactant (Triton X-100) = 1.5ml, sonicated for 30 min with a pulse of 5 sec on / off and centrifuge for 30 min, was filtered with polyvinylidene fluoride (PVDF) membrane having pore size of 0.22 µm.

**Figure S1.** As the MWCNT content increased, the thickness of the buckypaper and its resistivity increased. The minimum thickness of buckypaper was of the order of 55 μm, which was achieved with levels of 90 mg MWCNT. Below MWCNT content of 90 mg, the buckypaper broke during peeling off from the membrane.

**Figure S2.** At the same MWCNT content, the resistivity of buckypaper depends heavily on the amount of Triton X-100 (surfactant). It was observed that the resistivity of the buckypaper decreased when the content of Triton X-100 (surfactant) increased from 1.0 ml to 1.5 ml due to the better homogenization of the dispersion, but began to increase with the increase of the content of Triton X-100 from 1.5 ml to 2.0 ml due to trapping of surfactant inside the CNT network.

**Figure S3.** The resistivity of the buckypaper increases with the sonication time for 30 minutes. This shows that the CNTs were breaking into a smaller size after 30 minutes of sonication time.

**Figure S4.** The resistivity of the buckypaper decreased with increasing centrifugation time to 30 minutes and remained constant with increasing centrifugation time. The behavior indicated that all heavy particles had been removed from the dispersion after 30 minutes of centrifugation.

**Figure S5.** As the pore size of the membrane began to decrease, the micro gaps located between the CNT networks decreased, so that the resistivity decreased along with the pore size.

**Figure S6.** The membrane material and the quality of MWCNT have great influence on buckypaper resistivity.

**Table S1.** Summary table of Preprocessing Dispersion Optimization Process

| **Exp. Set** | **Vol of DI Water (ml)** | **MWCNT (mg)** | **Triton X-100 (ml)** | **Sonication Time (hrs)** | **Vol of Dispersion (ml)** | **Centrifuge Time (min)** | **Membrane Pore Size (um)** | **Membrane Material** | **MWCNT Quality** | **Sample Resistivity (ohm-mm)** |
| --- | --- | --- | --- | --- | --- | --- | --- | --- | --- | --- |
| 1 | 100 | 200 | 2.0 | 1 (Pulse 5sec) | 100 | 0 | 5 | PTFE | Local Supplier | 1.5125 |
|  |  | 150 |  |  |  |  |  |  |  | 1.4550 |
|  |  | 100 |  |  |  |  |  |  |  | 1.3261 |
|  |  | **90** |  |  |  |  |  |  |  | **1.2524** |
|  |  | 75 |  |  |  |  |  |  |  | Broke while Peeling |
| 2 | 100 | 90 | 2.0 | 1 (Pulse 5sec) | 100 | 0 | 5 | PTFE | Local Supplier | 1.2524 |
|  |  |  | **1.5** |  |  |  |  |  |  | **0.7387** |
|  |  |  | 1.0 |  |  |  |  |  |  | 1.0044 |
| 3 | 100 | 90 | 1.5 | **1 (Pulse 5sec)** | 100 | 0 | 5 | PTFE | Local Supplier | **0.7387** |
|  |  |  |  | 2 (Pulse 5sec) |  |  |  |  |  | 0.8233 |
|  |  |  |  | 3 (Pulse 5sec) |  |  |  |  |  | 0.9547 |
| 4 | 100 | 90 | 1.5 | 1 (Pulse 5sec) | 100 | 0 | 5 | PTFE | Local Supplier | 0.7387 |
|  |  |  |  |  | 120 | 10 |  |  |  | 0.4765 |
|  |  |  |  |  | **130** | **30** |  |  |  | **0.3799** |
|  |  |  |  |  | 140 | 60 |  |  |  | 0.3826 |
| 5 | 100 | 90 | 1.5 | 1 (Pulse 5sec) | 130 | 30 | 5 | PTFE | Local Supplier | 0.3799 |
|  |  |  |  |  | 90 |  | 1 |  |  | 0.3691 |
|  |  |  |  |  | 80 |  | 0.45 |  |  | 0.3536 |
|  |  |  |  |  | **70** |  | **0.22** |  |  | **0.3251** |
| 6 | 100 | 90 | 1.5 | 1 (Pulse 5sec) | 70 | 30 | 0.22 | PTFE | Local Supplier | 0.3251 |
|  |  |  |  |  |  |  |  | PES |  | 0.3116 |
|  |  |  |  |  |  |  |  | **PVDF** |  | **0.2249** |
|  |  |  |  |  |  |  | 0.2 | Nylon |  | 0.2589 |
|  |  |  |  |  |  |  |  | Al-Oxide |  | 0.2295 |
|  |  |  |  |  |  |  |  | MCE |  | 0.2886 |
|  |  |  |  |  |  |  |  | PC |  | 0.2655 |
| 7 | 100 | 90 | 1.5 | 1 (Pulse 5sec) | 70 | 30 | 0.22 | PVDF | Local Supplier | 0.2249 |
|  |  |  |  |  |  |  |  |  | **Foreign Supplier** | **0.1234** |

- **SCIM Process Flow**

***Pictorial Illustration of PI-Tape enabled Buckypaper Based Sensor Development***


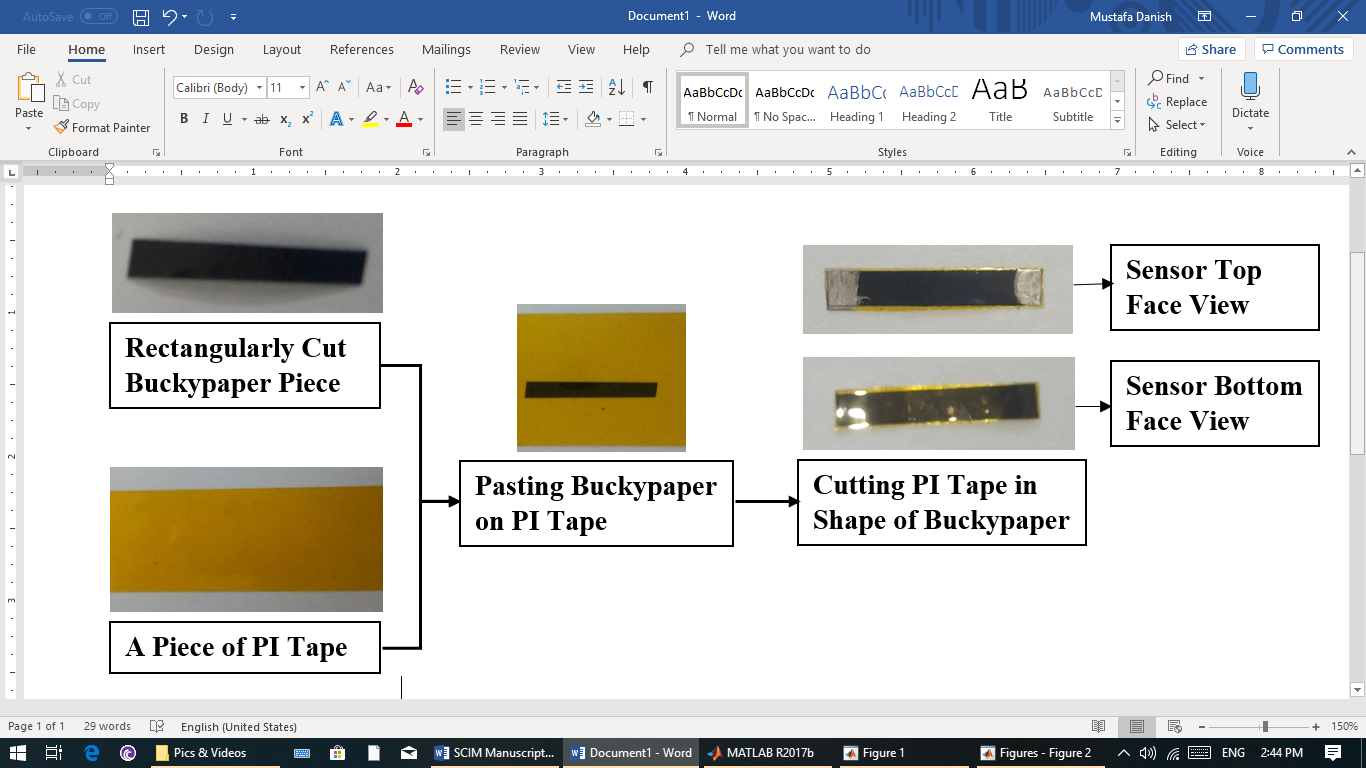


**Figure S7.** Pictorial block diagram illustration of PI-Tape enabled buckypaper based sensor.

***Block Diagram***

**Sensor Development**

**SCIM Implementation**

**Buckypaper Synthesize**

**Dispersion Preparation**

***Step-by-step sequence of key activities***


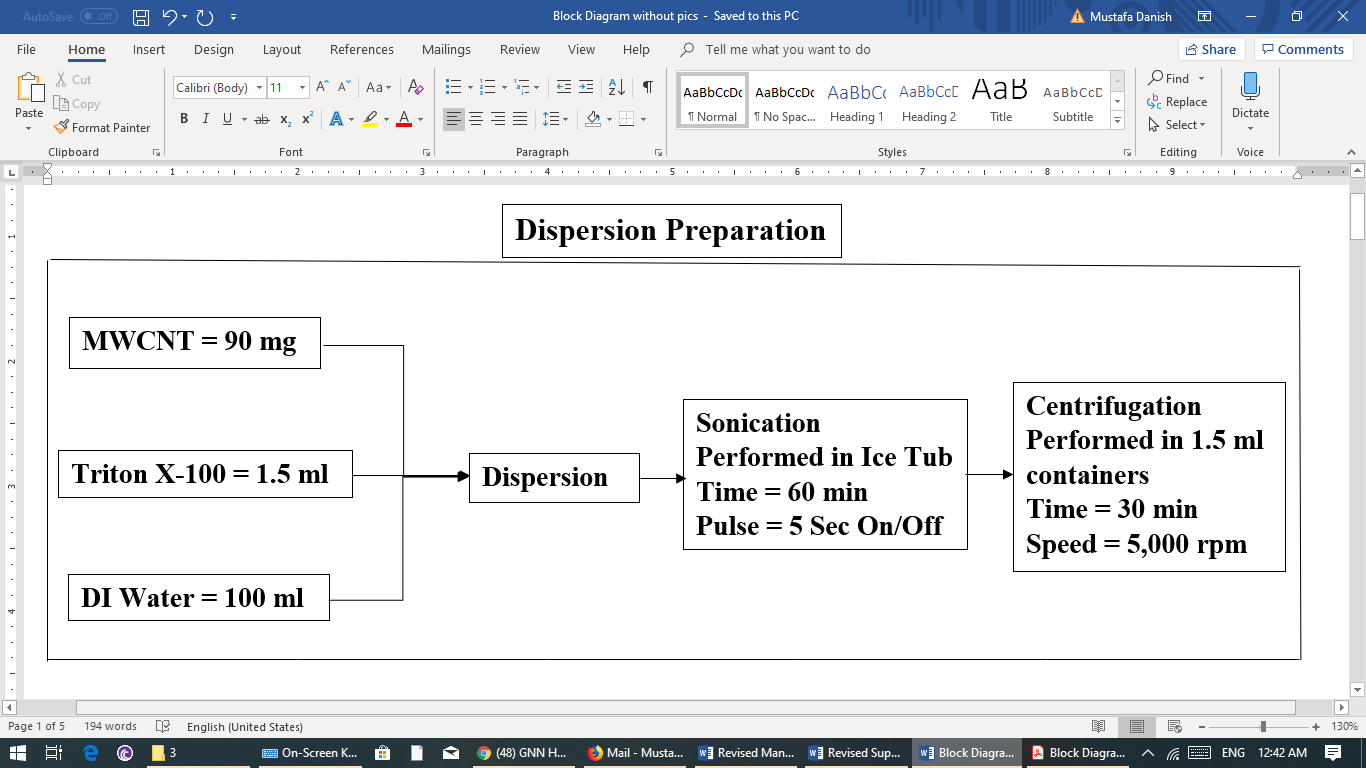


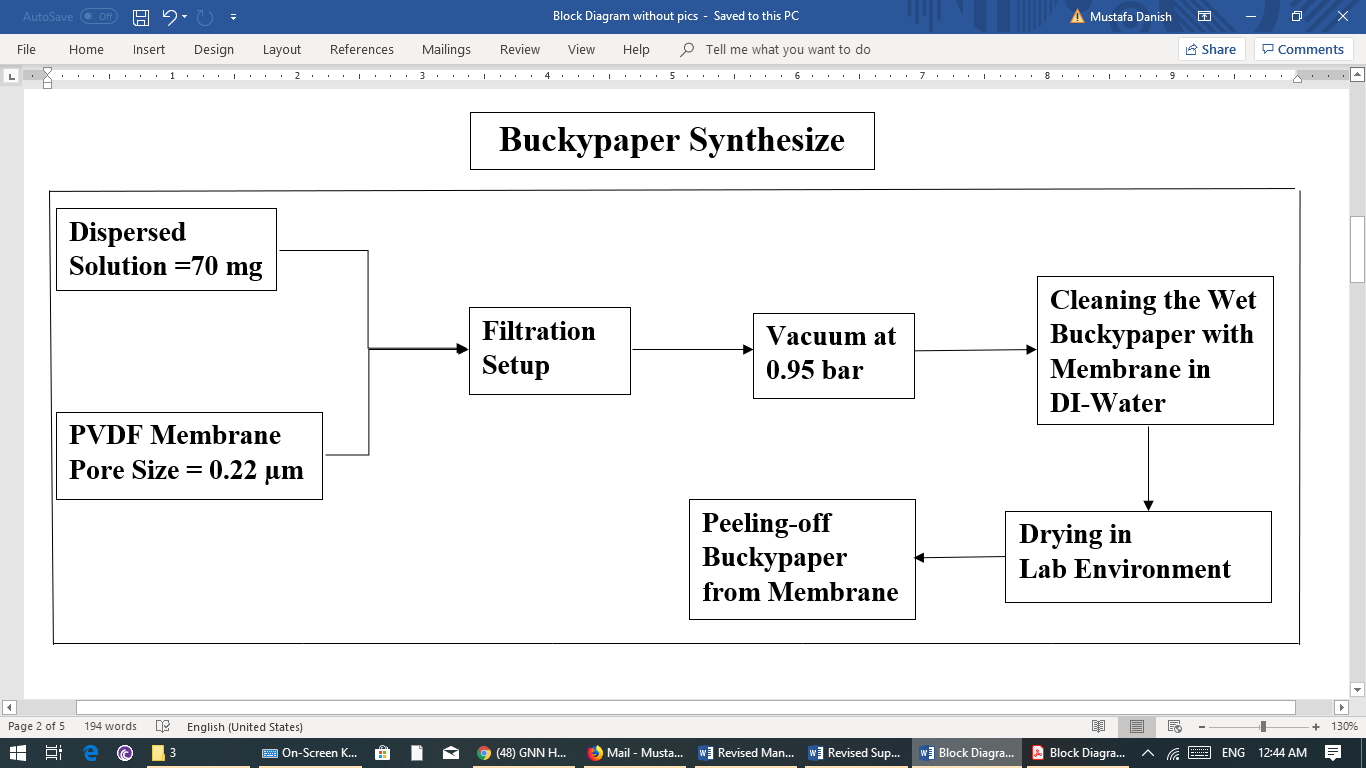


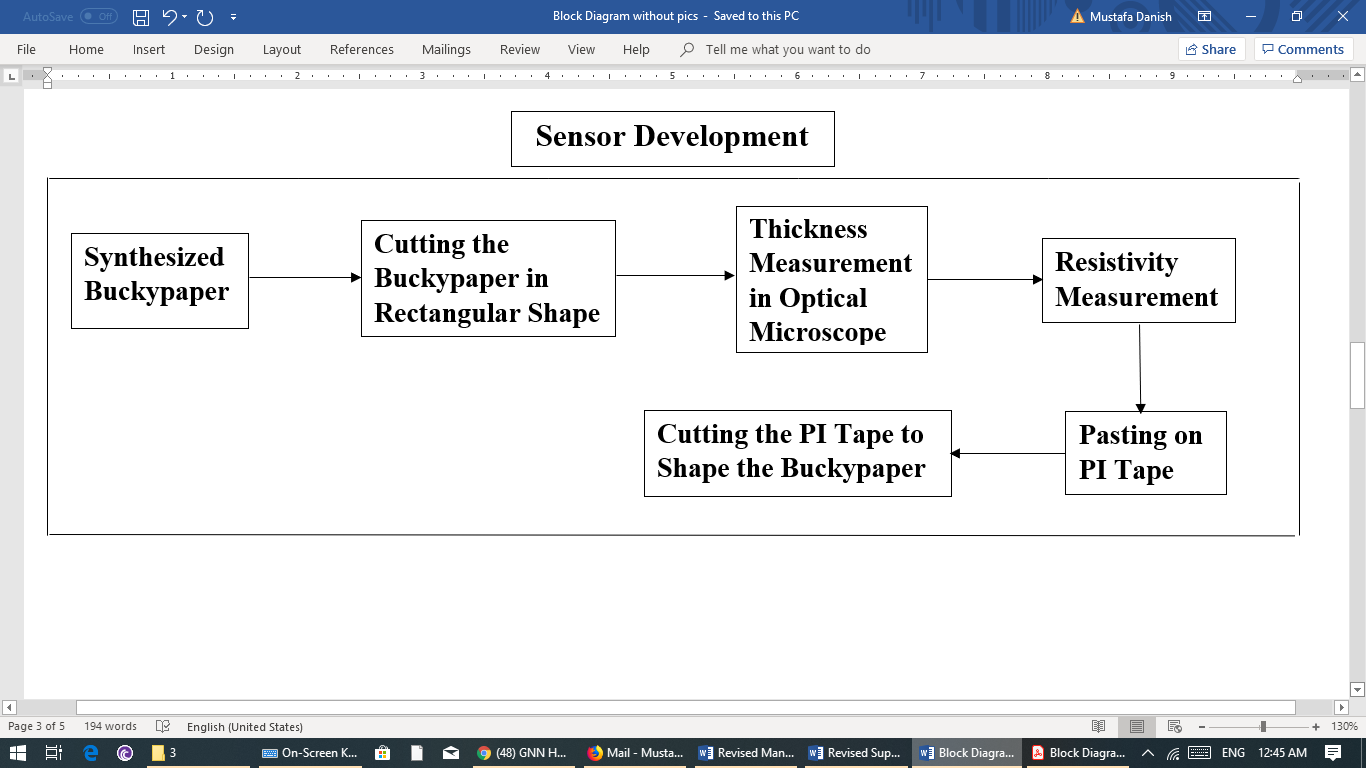


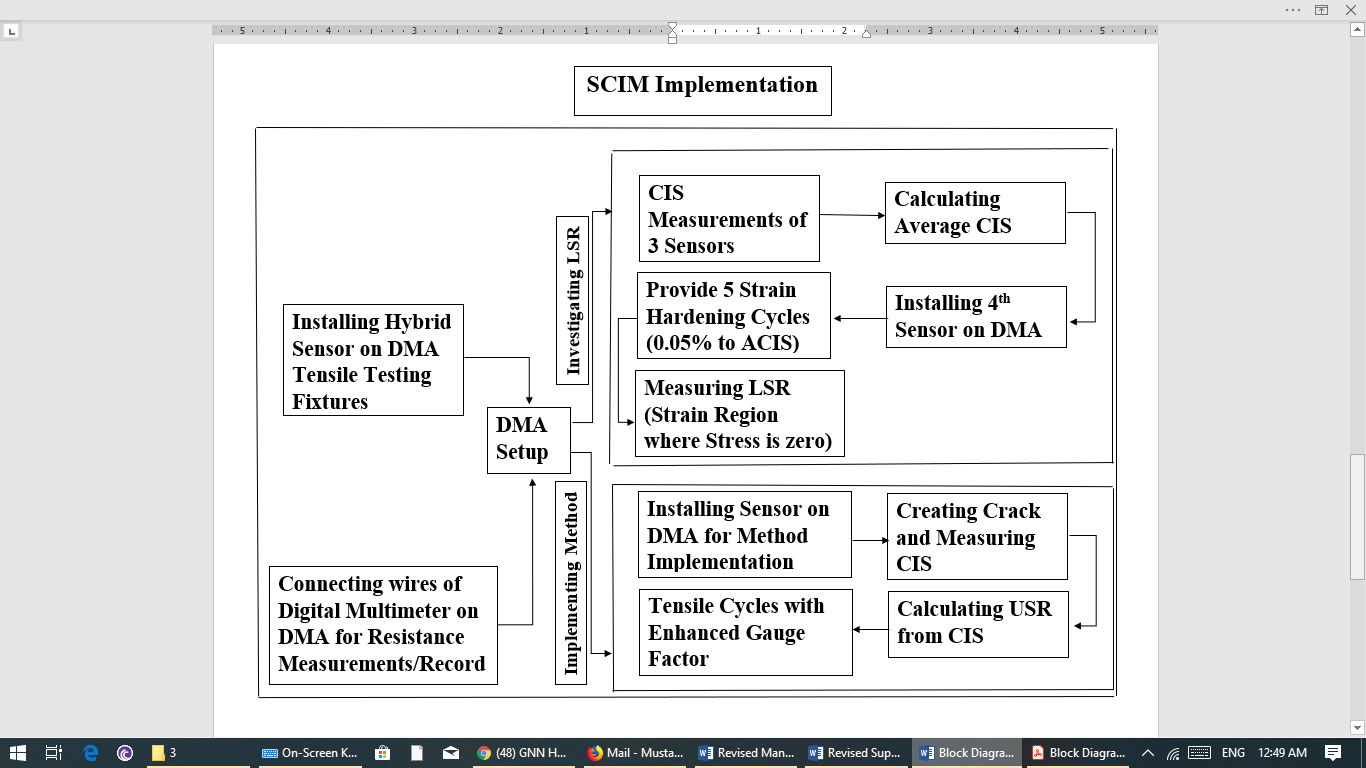


**Figure S8.** Detailed block diagram showing step-by-step sequence from dispersion preparation to SCIM implementation on sensor.

***Behavior of Stable and Unstable Cracks in Buckypaper Sensor***


 **(a)**  **(b)**

**Figure S9.** (a) Represents the change in resistance of the sensor in which a crack was generated by the implementation of the SCIM. The sensor has undergone tensile strain cycles between LSR and USR. The change in resistance remained the same during the strain cycles, and the lower and upper resistors remained constant, indicating that the crack remained stable and did not propagated. (b) Represents a change in resistance in the sensor in which a crack has been generated but propagated. The lower boundary of resistance remained constant, but the upper boundary of resistance increased continuously from one cycle to the next, suggesting that the crack opened and propagated with the cycles.

***Strain Hardening: Initial Tensile Cycle Behavior of PI-Tape Enabled Buckypaper Sensor***

**(a)**  **(b)**

**Figure S10.** (a) Represents the stress-strain diagram of the PI-tape enabled buckypaper based sensor. Initially, the curve shifted downwards and remained constant in the later cycles, indicating permanent deformation and strain loss, which in this study we call " strain hardening". (b) Represents the resistance change of the sensor when it is subjected to tensile strain cycles. A continuous increase in the lower and upper resistance values ​​was observed. The increase was intense during the initial cycles and then stabilized. The upper shift in resistance was also attributed to strain hardening, i.e., loss of strain or permanent deformation. This strain loss was taken into account by the LSR when the sensors were subjected to tensile cycles and included in the calculation of gauge factor and working strain region.
